# A Compact Wearable Textile Antenna for NB-IoT and ISM Band Patient Tracking Applications

**DOI:** 10.3390/s24155077

**Published:** 2024-08-05

**Authors:** Deepti Sharma, Rakesh N. Tiwari, Sachin Kumar, Satyendra Sharma, Ladislau Matekovits

**Affiliations:** 1 Department of Electronics and Communication Engineering, G L Bajaj Institute of Technology and Management, Greater Noida 201306, Uttar Pradesh, India; deeptidec24@gmail.com (D.S.); satyendracommn@gmail.com (S.S.); 2 Department of Electronics and Communication Engineering, Madanapalle Institute of Technology & Science, Madanapalle 517325, Andhra Pradesh, India; srakeshnath@gmail.com; 3 Department of Electronics and Communication Engineering, Galgotias College of Engineering and Technology, Greater Noida 201310, Uttar Pradesh, India; gupta.sachin0708@gmail.com; 4 Department of Electronics and Telecommunications, Politecnico di Torino, 10129 Turin, Italy; 5 Faculty of Electronics and Telecommunications, Politehnica University Timişoara, 300223 Timişoara, Romania; 6 Istituto di Elettronica e di Ingegneria dell’Informazione e delle Telecomunicazioni, National Research Council of Italy, 10129 Turin, Italy

**Keywords:** ISM (industrial scientific and medical) band, multilayer phantom model, NB-IoT (narrow band-internet of things), RFID (radio frequency identification), wearable antenna

## Abstract

This paper proposes a novel multi-band textile monopole antenna for patient tracking applications. The designed antenna has compact footprints (0.13*λ*_0_^2^) and works in the narrow band-internet of things (NB-IoT) 1.8 GHz, radio frequency identification (RFID), and industrial, scientific, and medical (ISM) 2.45 GHz and 5.8 GHz bands. The impedance bandwidths and gain of the antenna at 1.8 GHz, 2.45 GHz, and 5.8 GHz are 310 MHz, 960 MHz, and 1140 MHz; 3.7 dBi, 5.3 dBi, and 9.6 dBi, respectively. Also, the antenna’s behavior is checked on different body parts of the human body in various bending scenarios. As per the evaluated link budget, the designed antenna can easily communicate up to 100 m of distance. The specific absorption rate values of the designed antenna are also within acceptable limits as per the (FCC/ICNIRP) standards at the reported frequency bands. Unlike traditional rigid antennas, the proposed textile antenna is non-intrusive, enhancing user safety and comfort. The denim material makes it comfortable for extended wear, reducing the risk of skin irritation. It can also withstand regular wear and tear, including stretching and bending. The presented denim-based antenna can be seamlessly integrated into clothing and accessories, making it less obtrusive and more aesthetically pleasing.

## 1. Introduction

Globally, the number of patients is increasing owing to increasing numbers of diseases. Every year, around 421 million patients are hospitalized, out of which 42.7 million tragic deaths of patients happen due to medical errors alone [1]. Common medical errors are due to misidentification of patients’ specimens, inaccurate blood transfusion, and adverse drug events [2]. However, as per the Food and Drug Administration (FDA) estimation, half of the medical errors triggered by misidentification are avoidable [1]. Radio frequency identification (RFID) technology can prevent all the above-discussed issues in healthcare by providing patient tracking, medicine and tools tracking, and blood bank management.

A wearable textile antenna is proposed for patient tracking applications in the presented work. Although, in the literature, other flexible non-textile antennas are also proposed for wearable applications [3], the wearable antenna proposed here should seamlessly fit in garments for the wearer’s comfort. In this sense, textile antennas made from felt, denim, cotton, silk, etc., are a good choice for wearable applications. Flexible and stretchable antennas [4] are quite popular for different applications such as augmented and virtual realities [5], neonatal intensive care monitoring [6], and bio-integrated electronics [7].

A narrowband wearable textile antenna with an area of 50 mm × 50 mm was reported in [8]. It resonated in the 2.4 GHz and 5.2 GHz with −3.2 dBi and 6.6 dBi gain values, respectively. A dual ISM band planar inverted-F antenna (PIFA) textile antenna working at 433 MHz and 2.4 GHz was proposed in [9]. This antenna had −0.6 dBi and 6.8 dBi gain values and impedance bandwidths of 8.0% and 12.6% at the reported resonances (433 MHz and 2.45 GHz), respectively. Another textile-based PIFA antenna was proposed in [10]. This antenna had a gain of 6.7 dBi and worked only in a single 5 GHz frequency band for WBAN applications. In [11], a 71 mm × 65 mm size Yagi-like textile antenna for RFID applications was proposed. A defective ground wearable antenna with 104.7 mm × 100 mm with multiband behavior was reported in [12]. For wearable internet of things (IoT) applications, a tri-band antenna was proposed in [13]. This antenna had a size of 80 mm × 80 mm and it was functioning in the 2.4 GHz, 5.2 GHz, and 5.75 GHz frequency bands. In [14], wearable antenna for virtual reality was proposed, which worked at an 8 GHz frequency band. A wearable dual-band multi-layer patch antenna was proposed in [15]. However, this work did not evaluate specific absorption rate (SAR) and bending analysis.

Another wearable antenna for brain monitoring applications was reported in [16]. This antenna was non-textile with footprints of 70 mm × 30 mm and operated in the ISM 2.4 GHz frequency band. A low-profile textile-based antenna system was presented in [16] for body temperature and sweat sensing with an antenna size of 68 mm × 68 mm. Another low-profile multi-band textile circular patch antenna was reported in [17]. A compact single-band antenna (ISM 5.8 GHz) with a limited ground plane and low SAR value was proposed in [18].

A wearable textile quasi-Yagi antenna functioning in dual frequency bands (0.868 GHz and 2.45 GHz) was proposed in [19]. The area of this antenna was 65 mm × 60 mm, and the gain was relatively low (−1.4 dBi). A high-gain and high-profile (multi-layer substrate) textile antenna working in dual frequency bands (2.45 GHz and 3.45 GHz) of area 0.49*λ*_0_ × 0.49*λ*_0_ was reported in [20]. A narrowband and low-gain textile antenna working in multiple frequency bands was presented in [21]. One more antenna designed for military applications, functioning at 8 GHz, was explained in [22]. However, this antenna had a large size and low gain of 5.2 dBi. A multiband textile antenna with the ultrawideband (UWB) was proposed in [23] with a gain of 7.2 dBi. A single ISM 2.4 GHz textile antenna was proposed for wearable applications [24]. The antenna’s gain was 5.4 dBi, but the authors did not perform bending and SAR analyses. An ultra-high frequency (UHF) antenna for RFID applications was proposed in [25], and the antenna was embroidered on a surgical mask. This antenna showed wide bandwidth at the UHF frequency, which makes it suitable for wearable applications. Table 1 compares previously reported wearable textile antennas with the proposed antenna.

At present, healthcare technology requires wide bandwidth multiband antennas with optimum on-body gain for effective communication to the outside-body devices in all bending scenarios. Another textile antenna of size 0.43*λ*_0_ × 0.43*λ*_0_, working in the single frequency band (2.45 GHz), was explained in [26]. This antenna had a gain of 6.3 dBi and a bandwidth of 6.6%. One more antenna for RFID tags in the UHF band was proposed in [27] and worked only in the single resonance with a peak gain of −7.1 dBi. Embroidery-based antennas are also a decent option for wearable applications. However, all the embroidery antennas have some limitations, such as antennas being unable to use high-resolution designs, conducting threads used to realize the antenna having high resistivity compared to metal materials, and parasitic capacitances forming between the threads. Due to these reasons, some characterizing and optimizing techniques were proposed [28] to improve the behavior of these antennas. In order to obtain stable behavior from the wearable antennas, they must have the following properties, as discussed in [29]:

- The antenna should withstand different physical impacts related to regular usage;
- Multiband behavior is needed to support numerous applications;
- The wearable antenna must be small-sized, flexible, lightweight, and comfortable on the body.

Wearable antennas discussed in [10,11,25,27] were single-band antennas, and the antenna discussed in [11] was a single-band large-sized antenna. Multiband antennas were reported in [8,9,12,13,21], but antennas proposed in [8,9,21] had limited/narrow impedance bandwidth and antennas in [12,13] had large footprints. In [21], the gain in all the reported frequency bands was on the lower side, and the authors did not perform a bending analysis of the antenna to prove its usefulness in a natural environment. The high-profile antennas (multi-layer) were discussed in refs. [20,26]. Also, the antenna designed in ref. [26] is a single-frequency band antenna; however, the wearable antennas have to communicate to implants and external antennas, so it is necessary to have at least dual-band functioning. However, wide impedance bandwidth causes the antenna’s frequency bands to be unaffected by detuning caused by bending due to the curvature of the human body and its movement. However, the research work proposed in [19,23,27] did not consider SAR analysis, a critical parameter in the design of antennas for wearable applications. It identifies the safe input power value given to the antenna while working on the wearer’s body. Therefore, a compact, multiband antenna with good gain and wideband behavior must be designed. The multiband antenna enables data transfer, power transfer, and control signaling or data transfer at multiple data rates or can support different frequency applications. Also, a high-gain and wideband antenna enables wide-range tracking and makes the antenna immune to detuning in various bending environments.

**Figure 1:**
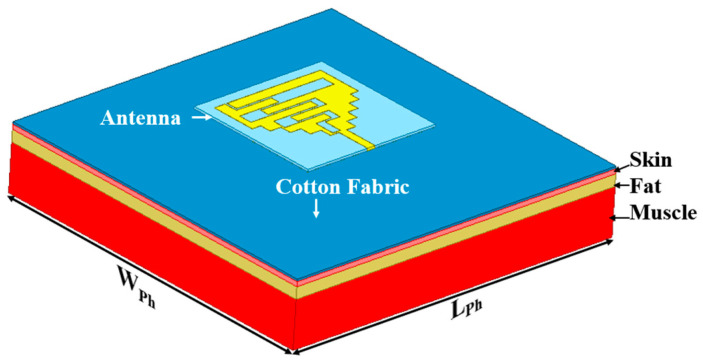
Wearable textile monopole antenna on phantom model (*W_Ph_* = *L_Ph_* = 150 mm).

Thus, keeping all the above factors in mind, a wearable textile monopole antenna is proposed on a denim fabric substrate with a volume of 60 mm × 60 mm × 1 mm for patient tracking in this paper. The proposed antenna works in the narrow band-internet of things (NB-IoT), RFID, and ISM 2.45 GHz/5.8 GHz frequency bands. NB-IoT technology offers long-range communications at a low-data rate for wearable sensors with a long battery life [30]. Meanwhile, 2.45 GHz and 5.8 GHz bands are used for RFID patient tracking applications because these bands are entirely safe for the human body, as per the Federal Communications Commission (FCC). The impedance bandwidths at 1.8 GHz, 2.45 GHz, and 5.8 GHz are 330 MHz, 780 MHz, and 900 MHz, respectively. Therefore, the proposed multiband antenna can use one frequency band for patient tracking (ISM RFID 2.4/5.8 GHz), and the other frequency band can support different applications. Also, the on-body gain values of the antenna are 3.7 dBi, 5.3 dBi, and 9.6 dBi, at 1.8 GHz, 2.45 GHz, and 5.8 GHz, respectively. The proposed antenna is designed on the multi-layer cuboid phantom to be compatible with patient tracking, with the thickness of skin, fat, and muscle to be 4 mm, 8 mm, and 25 mm, respectively [31], as shown in Figure 1. Each layer has dielectric properties as per Ref. [32].

The antenna’s geometry is a stepped square planar monopole antenna with asymmetric meandering slots. The ground plane is partial with slots and stubs, which helps to create boresight radiation in all the reported frequency bands. The safety of the wearable antenna is ensured by evaluating SAR on the multi-layer cuboid phantom body model, which is within the safety limits as per the FCC and ICNIRP.

To experimentally verify the behavior of the designed wearable antenna on the human body, its performance on different body parts such as the chest, arm, and wrist is checked. The antenna’s performance in all the wearable conditions is found satisfactory as it covers all the frequency bands even after little detuning due to bending.

## 2. Antenna Design Methodology

### 2.1. Antenna Design

**Figure 2:**
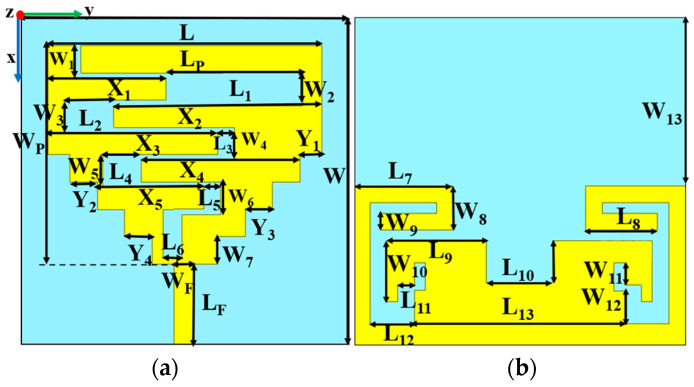
Design of antenna: (**a**) Top view (**b**) Bottom view.

The proposed multiband wearable antenna, depicted in Figure 2, is a stepped square planar monopole antenna with asymmetric meandering slots on top and backed in a partial and slotted ground plane. The antenna is designed on a denim substrate of permittivity (*ε_r_*) and loss tangent (tan *δ*) of 1.72 and 0.045, respectively. The Ansys HFSS 3D-electromagnetic tool was used for antenna design. The antenna’s total size is 60 mm × 60 mm; with detailed dimensions given in Table 2. Here, the lower NB-IoT 1.8 GHz frequency band is achieved by the stepped structure of the top patch. The asymmetric meandering slot on the top patch and slotted ground plane with stubs helped to achieve RFID ISM 2.45 GHz and 5.8 GHz with wideband behavior.

### 2.2. Evolution of the Antenna

**Figure 3:**
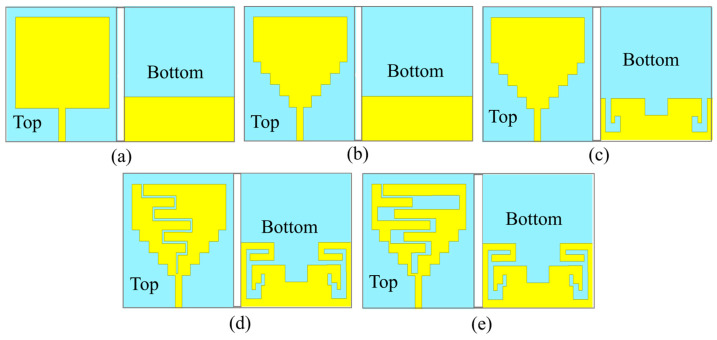
Design evolution of the antenna in five steps: (**a**) Step 0, (**b**) Step 1, (**c**) Step 2, (**d**) Step 3, (**e**) Step 4 (Proposed).

There are five steps of antenna evolution, as shown in Figure 3. Initially, a denim substrate of the size of 60 mm × 60 mm was chosen to design a square monopole patch antenna with microstrip feed and the partial ground plane (after placing it on cotton fabric of 3 mm thickness), as shown in Figure 3a, to reduce the dielectric loading due to high dielectric constant of biological tissues of human body [33].

**Figure 4:**
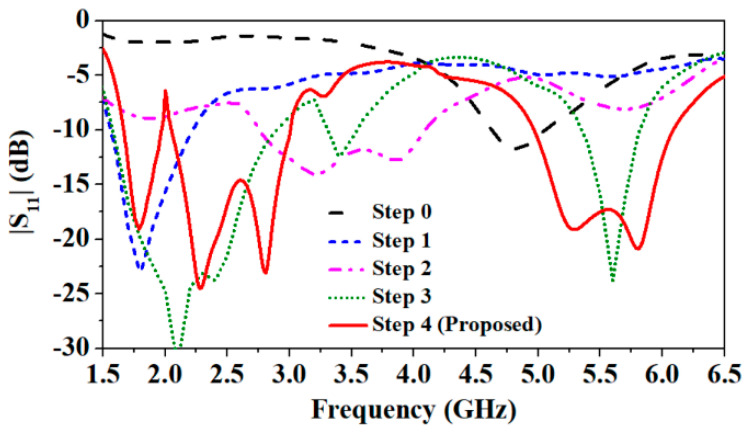
Simulated |S_11_| of design evolution.

**Figure 5:**
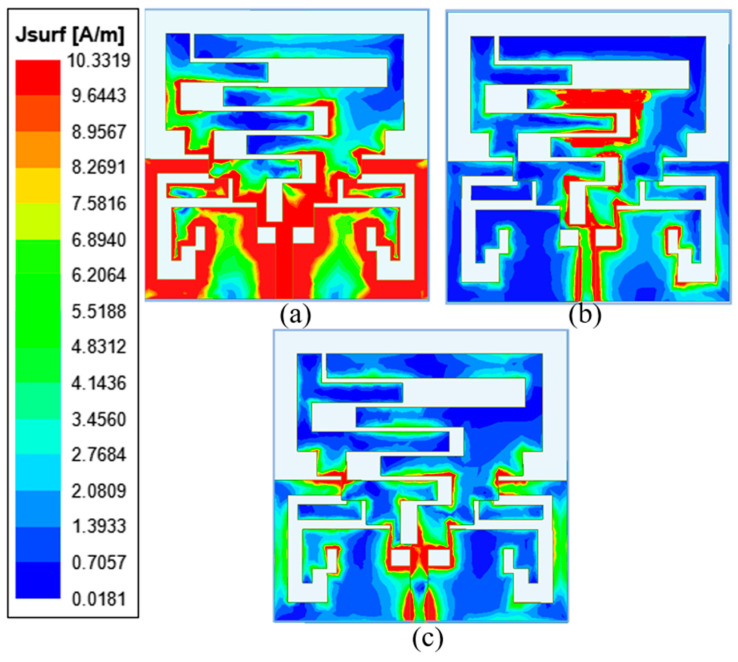
Current distribution at (**a**) 1.8 GHz, (**b**) 2.45 GHz, (**c**) 5.8 GHz.

In step 0 of antenna evolution, the antenna is resonating at 4.8 GHz, as shown in Figure 4 This change shifted the 4.8 GHz resonance to 1.8 GHz, due to increased current path along the width of the antenna, as shown in Figure 5. In the second step (step 1), four rectangular slots of the same width and different length along the width are etched, to create the stepped profile, as shown in Figure 3b.

Up to this point, the antenna is resonating in the single frequency band of 1.8 GHz, but the objective is to design a multiband antenna that could support multiple applications. Therefore, in the third step (step 2, Figure 3c), the rectangular slot is etched along the length at the center of the partial ground plane. Due to this, the current vectors split, and multiple frequency bands are created. Next, two L-shaped hook-like slots are created along the width of the partial ground plane that helped to increase the current path. Now, a second resonance at 3.2 GHz is obtained with wide impedance bandwidth and a third resonance at 5.7 GHz with poor impedance matching at 1.8 GHz and 5.7 GHz.

Next, in the fourth step (step 3, Figure 3d), an asymmetric meandering line is etched from the top of the patch, and two bent metal strips are also connected to each end of the ground plane. It increased the current path and caused a shift in the middle 3.2 GHz resonance to 2.3 GHz and the higher frequency band 5.8 GHz to 5.7 GHz. However, the middle and the high-frequency bands shifted to the lower side, but the lowest frequency band (1.8 GHz) shifted to a slightly higher side (2.1 GHz). One more resonance at 3.4 GHz is also noticed. In this step, a good impedance matching in all the resonating bands is achieved, but the desired bands needed to be tuned. Consequently, in the final step (step 4, Figure 3e), the asymmetric meandering of the top patch is modified, and the width of each positive and negative peak of meandering line slots is increased. Due to the modified meandering slots, a merger of two close resonances (2.45–2.8 GHz) and (5.1–5.8 GHz) is created at the second (2.45 GHz) and third (5.8 GHz) ISM frequency bands, respectively. Finally, resonances at 1.8 GHz NB-IoT band, RFID ISM 2.45 GHz, and 5.8 GHz bands are achieved with impedance bandwidths of 0.31 GHz (1.68–1.97 GHz), 0.96 GHz (2.05–3.01 GHz), and 1.14 GHz (4.96–6.1 GHz), respectively.

Current distributions at 1.8 GHz, 2.45 GHz, and 5.8 GHz on the proposed antenna are shown in Figure 5. From Figure 5a it is clear that the antenna’s stepped section and ground plane show the highest current intensity at 1.8 GHz. The antenna’s top meandering slot and the ground plane’s slot created the ISM 2.45 GHz frequency band, which can be verified by high current intensity, as shown in Figure 5b. However, the rectangular ground slot helped to achieve the ISM 5.8 GHz frequency band, as shown in Figure 5c. The coupling between the human body and the wearable device is crucial to enhance antenna performance and minimize adverse effects on the body. Use of high-impedance surfaces can suppress surface waves, reducing the energy that couples into the body [33]. The coupling can also be reduced by placing a conductive shield between the antenna and the body.

### 2.3. Bending Analysis of Proposed Antenna

**Figure 6:**
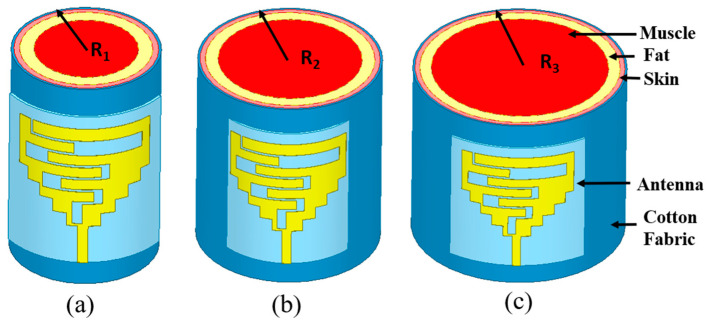
Analysis of bending states (at different radii) of the proposed antenna on cuboid phantom at (**a**) *R*_1_ = 25 mm, (**b**) *R*_2_ = 35 mm, (**c**) *R*_3_ = 45 mm.

**Figure 7:**
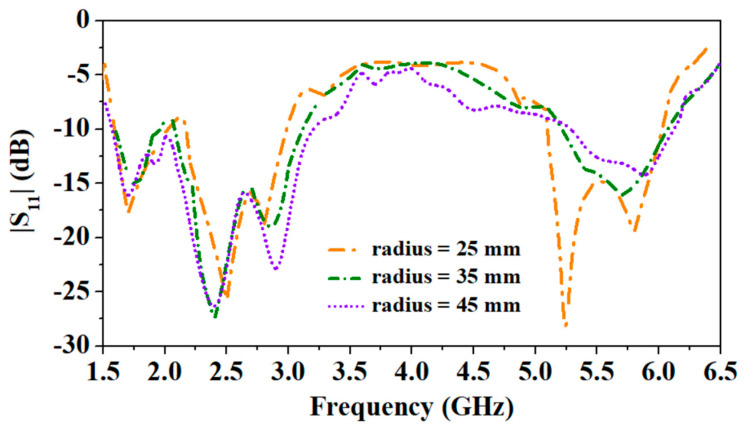
Comparison of |S_11_| at different radii of the phantom: 25, 35, and 45 mm.

The constraint for wearable antennas is physical conformability, which proves their capability to tolerate an evident bending and a convenient integration of antenna on the clothes of the human body [34]. The proposed antenna is designed to integrate into the wearable clothes of patients and can be placed on the human body’s chest, arm, or wrist. The average diameter of the arm for newborn babies, kids, and adults is 93 mm, 140 mm, and 350 mm, respectively [34]. Figure 6 and Figure 7 show different bending radii of the cuboid phantom body and corresponding results, respectively. Here, the thickness of the skin, fat, muscle, and cotton fabric is 2 mm, 5 mm, 13 mm, and 1 mm, respectively [31]. The results clearly show that due to the wideband nature of the designed antenna, it can easily handle a minimum bending of 25 mm, while the wrist of a newborn is not more than 35 mm.

Consequently, reflection coefficients at different curvature radii (25 mm, 35 mm, and 45 mm) of the antenna are shown in Figure 7. It can be noticed that the antenna experiences detuning when placed at the cylindrical multi-layer phantom. When the antenna was placed at a 25 mm radius phantom, it experienced maximum bending, and all the reported resonances shifted to the lower side.

At 35 mm and 45 mm radii, the antenna experiences less detuning. Since the designed antenna is wideband, it covers all the frequency bands (1.8 GHz, 2.45 GHz, and 5.8 GHz). Even after a little detuning due to the bending, this antenna is a suitable candidate for wearable applications due to its endurance to the bending at different radii.

### 2.4. Effect of the Human Body on the Antenna’s Behavior

**Figure 8:**
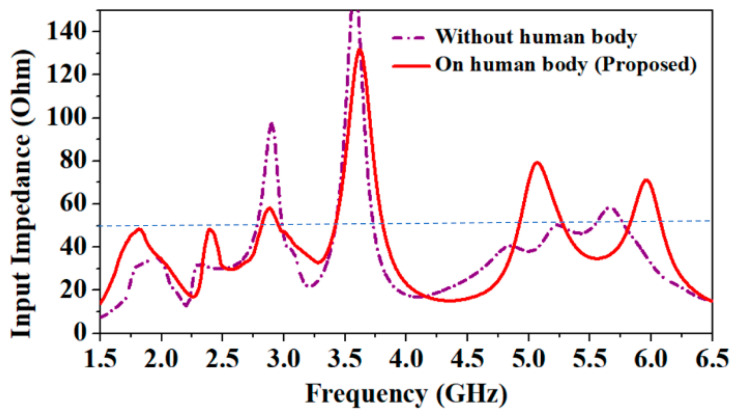
Comparison of antenna’s input impedance in the air (without the human body) and on the human body.

Here, the influence of the human body on the antenna’s parameters is shown. For this purpose, the input impedance of the antenna in air and on the human body are compared, as shown in Figure 8. Since the proposed antenna is optimized for wearable applications, it shows good input impedance matching (close to the 50 Ω) in all the operating frequency bands.

**Figure 9:**
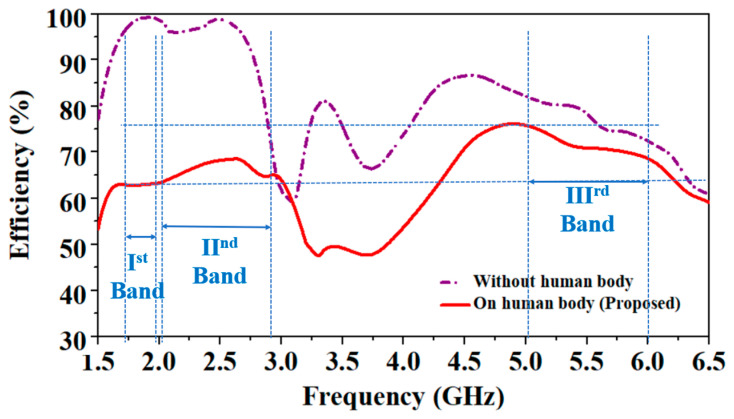
Comparison of antenna’s efficiency in the air (without the human body) and on the human body.

However, when its behavior was observed without a human body, it did not show good impedance matching in the lower frequency bands (1.8 GHz and 2.45 GHz). Since the human body is a lossy conductor and has a high dielectric constant compared to the denim substrate, its radiation efficiency is reduced due to the loading effect of the human body [33]. As shown in Figure 9, at low frequencies (1.8 GHz and 2.45 GHz), biological tissues have high permittivity compared to at high frequency, so the antenna faces more losses, and its efficiency becomes lower than at the high frequency (5.8 GHz). In the absence of the human body, the antenna shows maximum radiation efficiencies of 98.4%, 98.7%, and 75.1% at 1.8 GHz, 2.45 GHz, and 5.8 GHz frequencies, respectively. However, in the presence of the human body, efficiencies are 62.5%, 67.9%, and 70.1%, at 1.8 GHz, 2.45 GHz, and 5.8 GHz frequencies, respectively.

### 2.5. Antenna’s Performance at Different Distances from the Human Body

**Figure 10:**
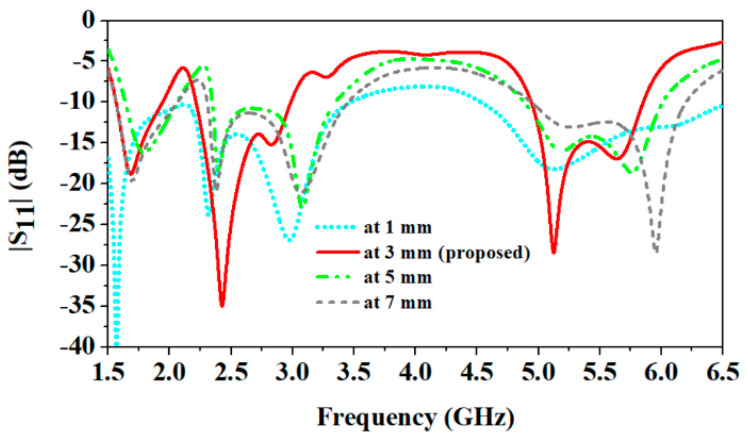
Comparison of antenna’s |S_11_| parameters at different distances from human body.

In this section, the impact of the antenna is investigated on different parameters, such as reflection coefficients and peak gain, when it is placed at various distances from the human body. At increasing distance values between the antenna and the human body, all the resonant frequencies shift to the higher side, as shown in Figure 10.

**Figure 11:**
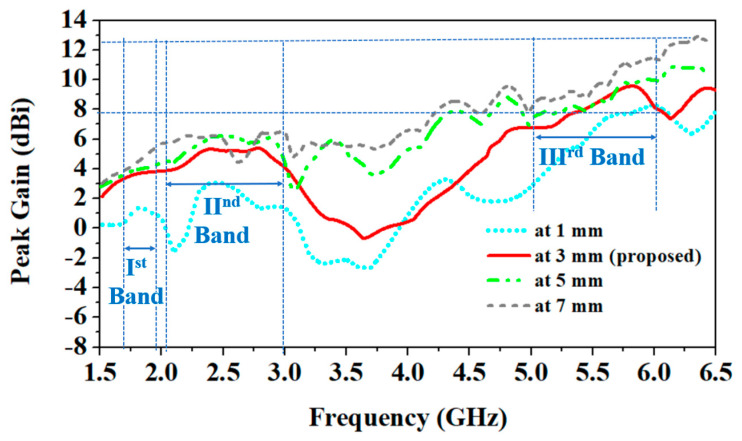
Comparison of antenna’s peak gain at different distances from human body.

Because the antenna moves away from the body, the impact of the human body’s high dielectric constant on the antenna reduces. Similarly, the high dielectric constant and conductivity of the biological tissues of the human body increase losses, and due to this, the antenna’s peak gain is reduced. As shown in Figure 11, as the distance between the antenna and the human body increases, the peak gain increases [33].

At a 1 mm distance between the antenna and the body, peak gain values at 1.8 GHz, 2.45 GHz, and 5.8 GHz are 1.6 dBi, 3.0 dBi, and 7.6 dBi, respectively. But, at 7 mm distance, peak gain values at 1.8 GHz, 2.45 GHz, and 5.8 GHz are 4.4 dBi, 6.3 dBi, and 10.9 dBi, respectively.

## 3. Results and Discussion

### 3.1. Reflection Coefficient Measurements

**Figure 12:**
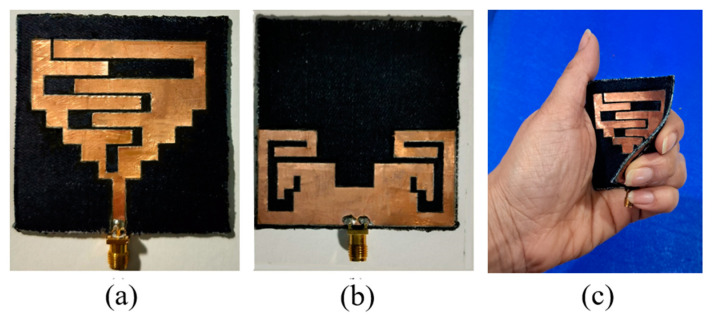
Antenna’s fabricated prototype: (**a**) Top, (**b**) Bottom, (**c**) In a random conformal state.

**Figure 13:**
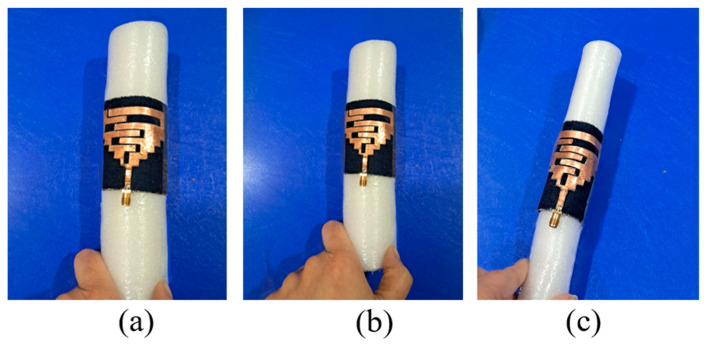
Antenna’s bending at different radii (in air): (**a**) 45 mm, (**b**) 35 mm, (**c**) 25 mm.

**Figure 14:**
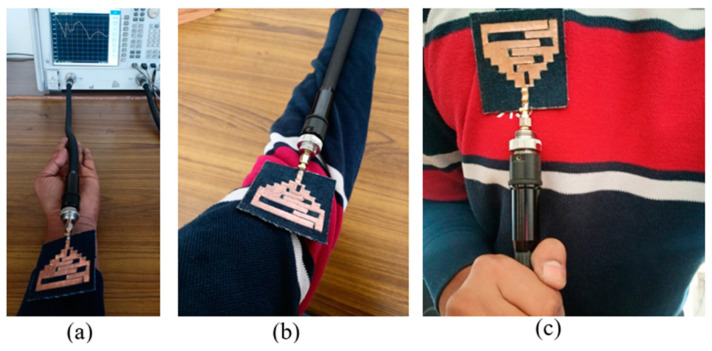
Antenna’s measurement: (**a**) On wrist, (**b**) On arm, (**c**) On chest.

**Figure 15:**
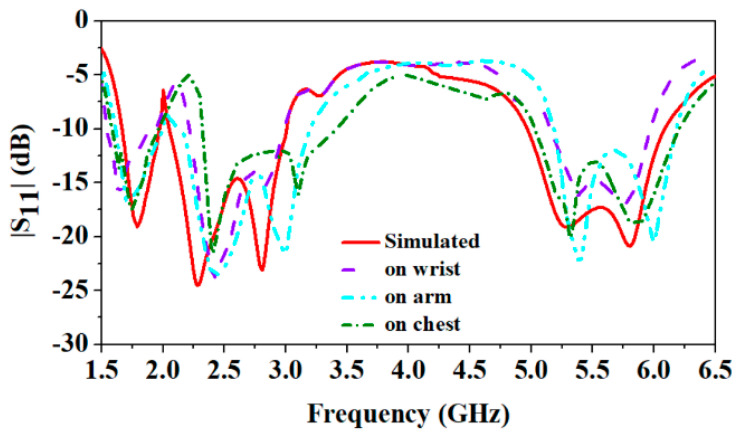
Comparison of simulated and measured reflection coefficients.

The proposed antenna is fabricated on the denim textile fabric, as shown in Figure 12. The antenna’s bending on the different values of radius (45 mm, 35 mm, and 25 mm) for the experimental verification is shown in Figure 13. Also, the antenna’s performance is measured by placing it on the wrist, arm, and chest, are shown in Figure 14. Furthermore, a comparison of simulated and measured reflection coefficients is shown in Figure 15.

It can be observed that the antenna covers all the reported frequency bands when placed on the different body parts; only a slight detuning is observed and that also does not affect the performance due to wide bandwidths in all the reported frequency bands.

**Figure 16:**
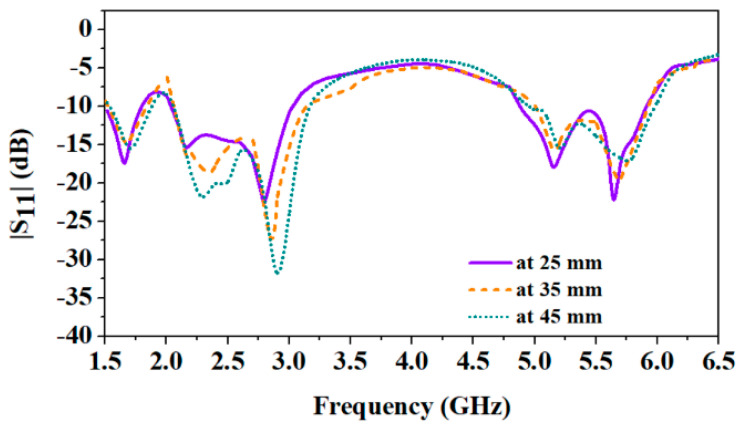
Comparison of measured reflection coefficients in different bending scenarios (at 25 mm, 35 mm, and 45 mm).

Also, reflection coefficients in different bending scenarios (as shown in Figure 14) are shown in Figure 16. It can be observed that the proposed antenna shows a lower side shift of resonant frequencies as it experiences more bending. A small bending radius causes a shift of resonant frequencies on the lower side [27]. But, all the reported frequency bands are still covered effectively, owing to the wideband behavior of the proposed wearable antenna.

### 3.2. Radiation Pattern Measurements

**Figure 17:**
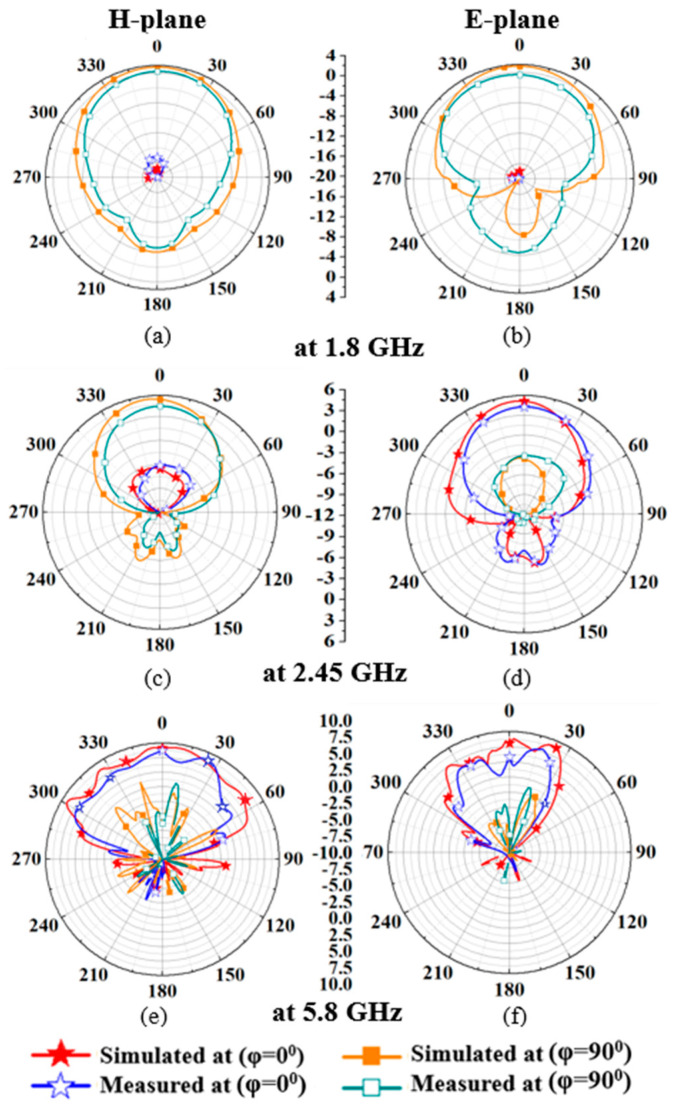
Comparison of simulated and measured radiation patterns of the proposed antenna (**a**) H-plane at 1.8 GHz (**b**) E-plane at 1.8 GHz (**c**) H-plane at 2.45 GHz (**d**) E-plane at 2.45 GHz (**e**) H-plane at 5.8 GHz (**f**) E-plane at 5.8 GHz.

**Figure 18:**
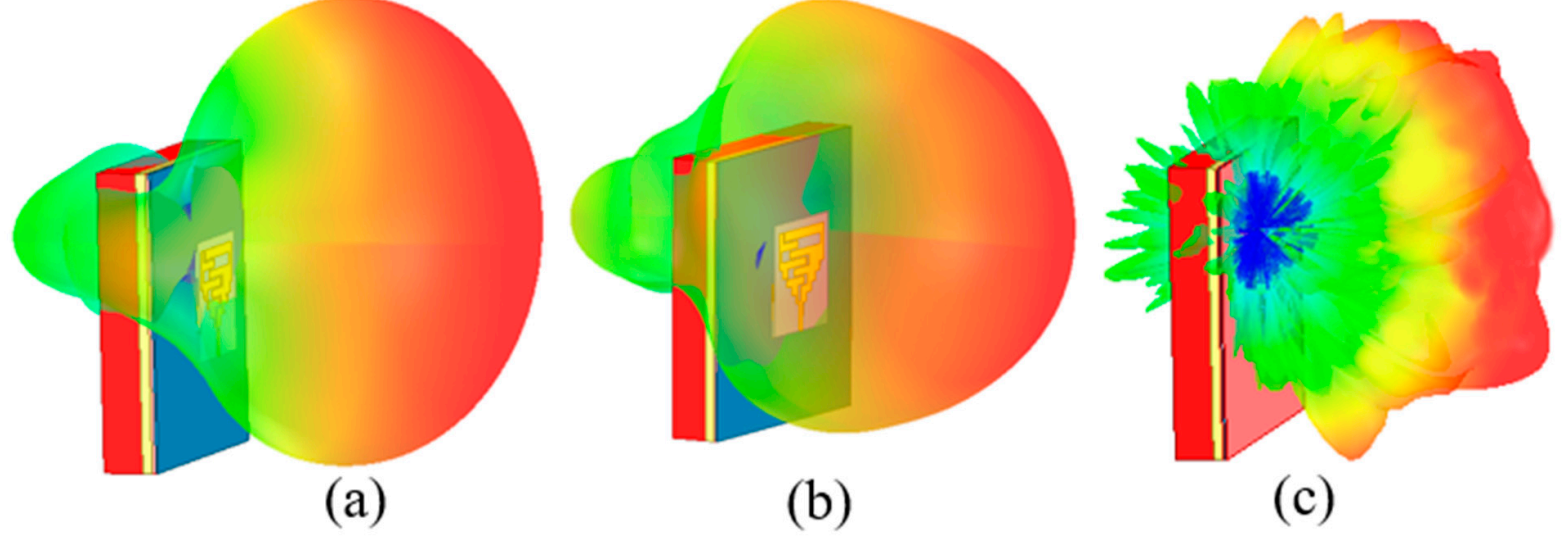
3D radiation patterns of the wearable textile antenna: (**a**) 1.8 GHz, (**b**) 2.45 GHz, (**c**) 5.8 GHz.

Figure 17a–f compares simulated and measured radiation patterns of the gain in the H-plane and E-plane. The simulated and measured peak gains are demonstrated in Table 3 (boresight direction (*θ* = 0°)). In Table 3, measured gain values are smaller than the simulated ones because the actual human body is larger than the cuboid phantom. At 1.8 GHz, according to Figure 17a,b, radiation patterns of the antenna in both the H (*ϕ* = 90˚) and E (*ϕ* = 90°) planes are nearly omnidirectional, and at 2.45 GHz, as shown in Figure 17c,d, most of the radiation is in boresight direction. However, at 5.8 GHz, the antenna shows a directive pattern (boresight), as shown in Figure 17e,f. Additionally, simulated 3D radiation patterns of the wearable textile antenna are shown in Figure 18a–c at 1.8, 2.45, and 5.8 GHz, respectively. Figure 18 clearly shows that the proposed textile antenna efficiently radiates outside the body at all three frequency bands (NB-IoT 1.8 GHz, RFID ISM 2.45 GHz, and 5.8 GHz), demonstrating its feasibility for stable wireless communication outside the body.

**Figure 19:**
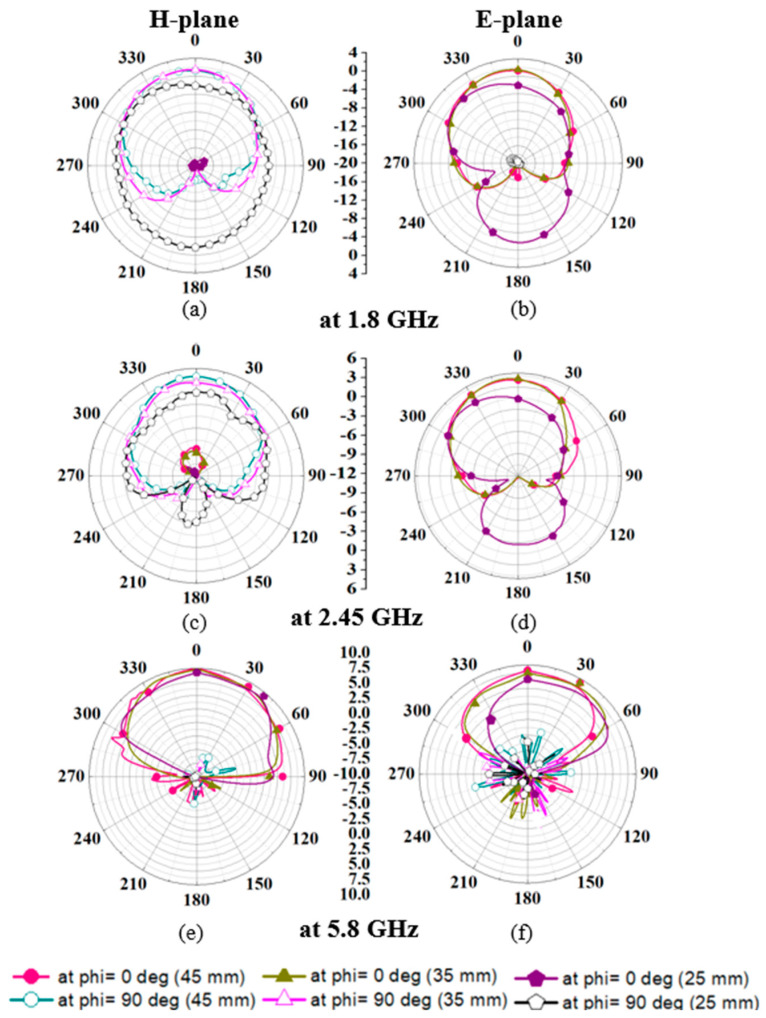
Comparison of measured radiation patterns in different bending scenarios (at 25 mm, 35 mm, and 45 mm) (**a**) H-plane at 1.8 GHz (**b**) E-plane at 1.8 GHz (**c**) H-plane at 2.45 GHz (**d**) E-plane at 2.45 GHz (**e**) H-plane at 5.8 GHz (**f**) E-plane at 5.8 GHz.

Subsequently, the radiation performance of the proposed antenna is measured in different bending scenarios, as shown in Figure 19a–f. When the antenna is bent in a 45 mm and 35 mm radius, the antenna radiated in a boresight direction. Similarly, when it is bent in a 25 mm radius, a slight tilt in the radiation pattern in the E-plane is observed, as shown in Figure 19a–f. Peak gain values in different bending scenarios are shown in Table 4.

### 3.3. Antenna’s Radiation Efficiency

**Figure 20:**
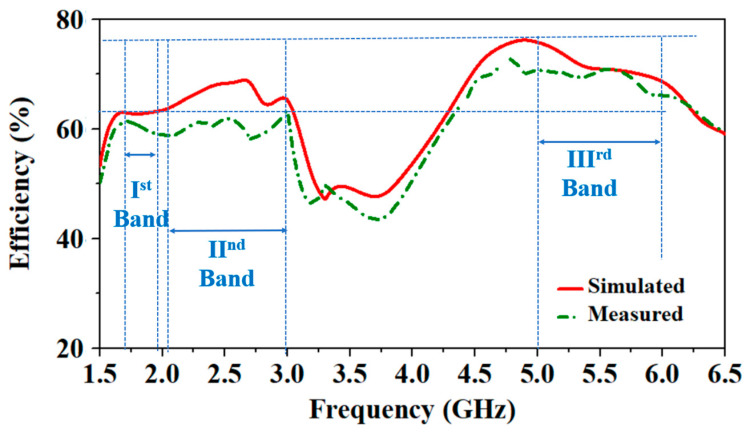
Simulated vs. measured efficiency of the proposed wearable antenna.

The simulated and measured antenna’s radiation efficiency values compared at the human chest are shown in Figure 20. The simulated radiation efficiencies at 1.8 GHz, 2.45 GHz, and 5.8 GHz are 62.5%, 67.9%, and 70.1%, respectively. The measured radiation efficiencies at 1.8 GHz, 2.45 GHz, and 5.8 GHz are 60.6%, 62.6%, and 68.3%, respectively. The measured values of radiation efficiencies are close to the simulated values; some differences might be due to the fabrication tolerances, the large size of the chest, and the comparatively small chest phantom in the simulator.

### 3.4. Specific Absorption Ratio (SAR) Analysis

**Figure 21:**
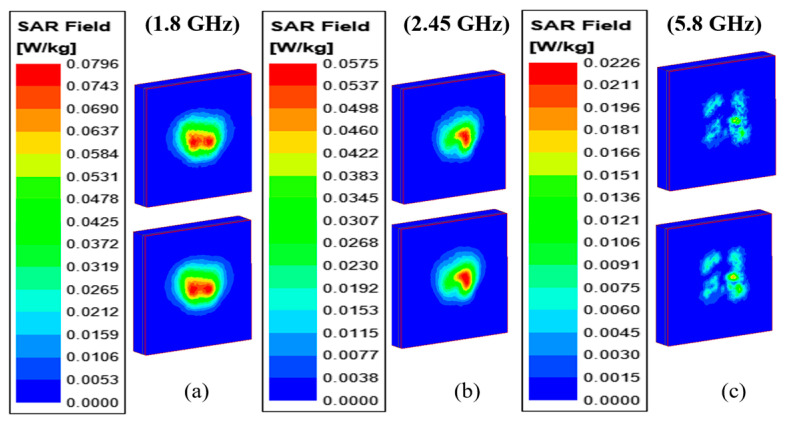
Simulated average SAR distribution on the cuboid phantom: at (**a**) 1.8 GHz (**b**) 2.45 GHz (**c**) 5.8 GHz.

Since the antenna is designed for patient tracking applications, it must be safe for long-term on-body operation. Therefore, evaluated SAR values are evaluated for 1 gm and 10 gm cubic tissue of the proposed wearable antenna on the multi-layer human body phantom from the standpoint of safety justification at the 1.8 GHz, 2.45 GHz, and 5.8 GHz frequencies, as shown in Figure 21. Human safety standards like ICNIRP guidelines and FCC [26] propose that the maximum average SAR (ASAR) value for 1 gm/10 gm of the cube of human tissue needs to be lower than the 1.6/2.0 W/kg. At 2.45 GHz and 5.8 GHz frequencies, the 1 gm and 10 gm ASAR (in W/kg) of the proposed antenna is shown in Table 5 (at 1 W of input power). At 1.8 GHz, 2.45 GHz, and 5.8 GHz, the 1 gm/10 gm average SAR values are 0.0796/0.0759, 0.0575/0.0552, 0.0226/0.0204, respectively. All the values of SAR are smaller than the standard values of 1.6 W/kg and 2.0 W/kg. In this case, 1 W of the input power is taken, but practically, all the wearable antennas need operating power in the mW range [35]. In that case, this antenna will have much smaller SAR values, which make it safe for body-worn applications.

### 3.5. Link Budget Analysis

In the present work, the communication capability of the designed wearable textile patient tracking antenna to the external monopole antenna with a gain of 2.15 dBi for off-body communication is explored. The Friis transmission equation is used to calculate the link budget [24,25] and Table 6 shows all the link budget parameters The antenna shows higher values of link margin at the lower frequencies (1.8 GHz and 2.45 GHz) and relatively lower values of link margin at high frequency (5.8 GHz) due to increased path losses at the higher frequency.

**Figure 22:**
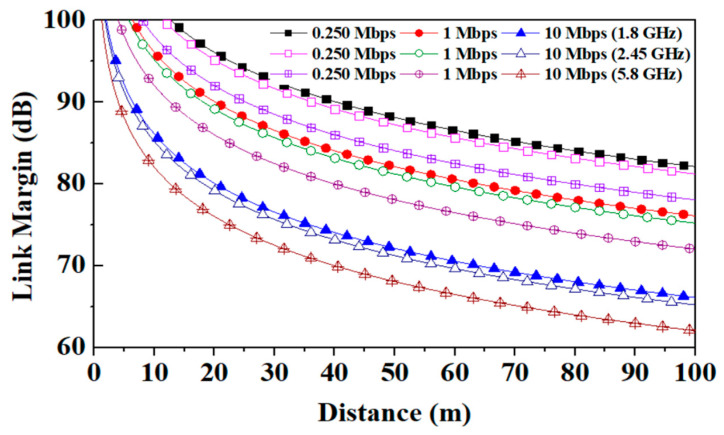
Link margin between *T_x_* (proposed ant.) and *R_x_* (monopole ant.) antennas at 1.8/2.45/5.8 GHz frequency bands.

However, Figure 22 shows that the proposed wearable antenna can bear additional losses of more than 62.5 dB when used for patient tracking up to 100 m at high frequency (5.8 GHz). The antenna’s communication link at all three reported frequency bands (1.8 GHz, 2.45 GHz, and 5.8 GHz) is good, and the proposed antenna can easily track up to 100 m.

## 4. Conclusions

A triple-band wearable textile monopole antenna is proposed for patient tracking applications. The designed antenna provides good gain (3.7 dBi, 5.3 dBi, and 9.6 dBi) and wide impedance bandwidths in all three reported frequency bands (1.8 GHz, 2.45 GHz, and 5.8 GHz). A bending analysis is performed to test the antenna’s usefulness in different bending scenarios. Owing to its wideband behavior, this antenna can operate in the reported frequency bands even if the antenna’s resonances shift. The proposed antenna radiates in a boresight direction (outside the human body) with wide impedance bandwidths and lower SAR values. It can communicate up to 100 m of distance, which makes it a suitable candidate for NB-IoT 1.8 GHz, ISM RFID 2.45 GHz, and 5.8 GHz wearable patient tracking applications.

## Figures and Tables

**Table 1:** Proposed antenna and previously reported textile antennas.

Ref. (Year)	[19] (2020)	[20] (2021)	[21] (2022)	[22] (2022)	[23] (2023)	This Work
Area (mm^2^)	65 × 60	60 × 60	60 × 60	55 × 40	84 × 69	60 × 60
Area (*λ*_0_^2^)	0.18 × 0.17 (0.03)	0.49 × 0.49 (0.24)	0.64 × 0.64 (0.41)	1.46 × 1.06 (1.55)	0.55 × 0.67 (0.37)	0.36 × 0.36 (0.13)
Frequency (GHz)	0.868/2.45	2.45/3.45	2.4/3.32/3.93/5.8	8	2.4/5	1.8/2.45/5.8
B.W. (%)	NG/3.5	4.9/6.7	3.7/5.7/ 5.85/9.8	13.1	5/76	17.2/39.1/19.6
Peak gain (dBi)	NG/−1.4	6.7/8.9	−0.81/−2.81/−1.16/2.8	5.2	7.2	3.7/5.3/9.6
SAR (W/Kg) 1 gm/10 gm	NG	0.1/0.04 (at 0.5 W)	0.11/0.33 (at 1 W)	0.7/--- (at 1 W)	NG	0.0796/0.0759 0.0575/0.0552 0.0226/0.0204 (at 1 W)

Note: NG—Not Given.

**Table 2:** Antenna’s design parameters (in mm).

Symbol	Value	Symbol	Value	Symbol	Value	Symbol	Value
*L*	60	*L*_7_	18	*W_F_*	3.6	*X*_1_	18.5
*L_P_*	50	*L*_8_	13	*W*_1_–*W*_6_	6.0	*X*_2_	34.5
*L_F_*	15	*L*_9_	18	*W*_7_	05	*X*_3_	21.0
*L*_1_	25	*L*_10_	12	*W*_8_	11	*X*_4_	17.0
*L*_2_	09	*L*_11_	3.0	*W*_9_	03	*X*_5_	7.5
*L*_3_	03	*L*_12_	08	*W*_10_	11	*Y*_1_	4.0
*L*_4_	07	*L*_13_	38	*W*_11_	03	*Y*_2_–*Y*_4_	5.0
*L*_5_	03	*W*	60	*W*_12_	06		
*L*_6_	3.5	*W_P_*	40	*W*_13_	31		

**Table 3:** Boresight peak gain values (dBi).

Frequency (GHz)	Simulation (Chest Phantom)	Measured (on Human Chest)
1.8	3.7	2.8
2.45	5.3	4.6
5.8	9.6	8.2

**Table 4:** Boresight peak gain values (dBi) at different bending radii: 45 mm, 35 mm, and 25 mm.

Frequency (GHz)	At 45 mm	At 35 mm	At 25 mm
1.8	2.1	2.0	0.2
2.45	4.5	4.6	3.2
5.8	8.2	8.1	7.8

**Table 5:** Maximum SAR of the proposed antenna (at 1 W input power).

Frequency (GHz)	Maximum SAR (on Phantom)	
	1 gm	10 gm
1.8	0.0796	0.0759
2.45	0.0575	0.0552
5.8	0.0226	0.0204

**Table 6:** Link budget parameters.

Transmitter		
	Frequency (GHz)	1.8/2.45/5.8
*G_t_*	Antenna gain (dBi)	3.7/5.3/9.6
*P_t_*	Transmitted power (dBm)	16
	EIRP (dBm)	19.7/21.3/25.6
**Receiver**		
*G_r_*	Receiver antenna gain (dBi) (external antenna)	2.15
*T_o_*	Ambient temperature (K)	293
	Boltzmann constant	1.38 × 10^−23^
*N_o_*	Noise power density (dB/Hz)	−203.9
**Signal quality**		
*B_r_*	Bit rate (Mbps)	0.250, 1, 10
*E_b_*/*N_o_*	Ideal PSK (dB)	9.6
*G_c_*	Coding gain (dB)	0
*G_d_*	Fixing deterioration (dB)	2.5

## Data Availability

Complete data is available in the research paper.
